# Autocrine hGH stimulates oncogenicity, epithelial-mesenchymal transition and cancer stem cell-like behavior in human colorectal carcinoma

**DOI:** 10.18632/oncotarget.21812

**Published:** 2017-10-10

**Authors:** Jing-Jing Wang, Qing-Yun Chong, Xin-Bao Sun, Ming-Liang You, Vijay Pandey, Yi-Jun Chen, Qiu-Shi Zhuang, Dong-Xu Liu, Lan Ma, Zheng-Sheng Wu, Tao Zhu, Peter E. Lobie

**Affiliations:** ^1^ Cancer Science Institute of Singapore and Department of Pharmacology, National University of Singapore, Singapore, Singapore; ^2^ Hefei National Laboratory for Physical Sciences at Microscale, University of Science and Technology of China, Hefei, China; ^3^ The CAS Key Laboratory of Innate Immunity and Chronic Disease, School of Life Sciences, University of Science and Technology of China, Hefei, China; ^4^ School of Science, Auckland University of Technology, Auckland, New Zealand; ^5^ Tsinghua Berkeley Shenzhen Institute, Tsinghua University Graduate School at Shenzhen, Shenzhen, China; ^6^ Department of Pathology, Anhui Medical University, Hefei, China; ^7^ National University Cancer Institute, Singapore, Singapore

**Keywords:** growth hormone, ERK, epithelial-mesenchymal transition, cancer stem cell, colorectal carcinoma

## Abstract

Tumor derived human growth hormone (hGH) has been implicated in cancer development and progression. However, the specific functional role of autocrine/paracrine hGH in colorectal cancer (CRC) remains largely to be determined. Herein, we demonstrated a crucial oncogenic role of autocrine hGH in CRC progression. Elevated hGH expression was detected in CRC compared to normal colorectal tissue, and hGH expression in CRC was positively associated with tumor size and lymph node metastasis. Forced expression of hGH stimulated cell proliferation, survival, oncogenicity and epithelial to mesenchymal transition (EMT) of CRC cells, and promoted xenograft growth and local invasion *in vivo*. Autocrine hGH expression in CRC cells stimulated the activation of the ERK1/2 pathway, which in turn resulted in increased transcription of the mesenchymal marker FIBRONECTIN 1 and transcriptional repression of the epithelial marker E-CADHERIN. The autocrine hGH-stimulated increase in CRC cell proliferation, cell survival and EMT was abrogated upon ERK1/2 inhibition. Furthermore, autocrine hGH-stimulated CRC cell migration and invasion was dependent on the ERK1/2-mediated increase in FIBRONECTIN 1 expression and decrease in E-CADHERIN expression. Forced expression of hGH also enhanced CSC-like behavior of CRC cells, as characterized by increased colonosphere formation, ALDH-positive population and CSC marker expression. Autocrine hGH-enhanced cancer stem cell (CSC)-like behavior in CRC cells was also observed to be E-CADHERIN-dependent. Thus, autocrine hGH plays a critical role in CRC progression, and inhibition of hGH could be a promising targeted therapeutic approach to limit disease progression in metastatic CRC patients.

## INTRODUCTION

Colorectal cancer (CRC) remains one of the most common malignant diseases worldwide with high incidence and cancer-related mortality [1]. During the progression of CRC, the abnormal proliferation of the epithelial cells in the mucosal lining of the colorectum gives rise to the primary tumor, which acquires the invasive capacity to spread into surrounding tissues and metastasize to distant organs, resulting in worse clinical outcomes [2]. Despite advances in the screening and treatment of CRC, more than 50% of CRC patients eventually develop invasive or metastatic disease [3, 4], and the 5-year survival rate of the patients with metastatic CRC is less than 15% [5]. Hence, understanding the mechanisms of CRC progression is critical for the development of new therapeutic strategies for CRC patients with metastasis.

Epithelial to mesenchymal transition (EMT) is a process where epithelial cells lose their cell polarity, intercellular contact and cellular adhesion to the basement membrane, and begin to acquire mesenchymal phenotypes including increased cell migratory and invasive capacity [6]. EMT plays a fundamental role in embryonic development [7], adult tissue regeneration and repair [8], as well as cancer progression from epithelial tumors to metastatic carcinomas [9]. EMT is characterized by downregulation of epithelial proteins and upregulation of mesenchymal proteins [9, 10]. Among these proteins, the epithelial cell adhesion molecule (E-CADHERIN) is thought to be a key molecular marker of the epithelial state and loss of E-CADHERIN expression is postulated to be a prominent phenomenon during EMT [6]. Recently, an increasing number of studies have suggested that the cancer stem cell (CSC) phenotype is associated with EMT and metastasis, resulting in poor clinical outcomes in cancer patients [11–13]. CSCs are a subpopulation of cancer cells that possess both the capabilities of sustaining tumor growth and tumor initiation [14]. Recent studies in CRC have also demonstrated the capacity of CSCs to initiate tumor growth and induce distant metastasis [15–17]. This reinforces the need for a better understanding of the mechanisms involved in the promotion of EMT and acquisition of CSC properties in CRC to prevent tumor relapse.

Growth hormone (GH) acts in an endocrine manner to regulate postnatal growth through the stimulation of cell proliferation, differentiation and metabolism in target tissues [18, 19, 91]. Recently, the association of tumor derived human growth hormone (hGH) with cancer has been well established [20]. Previous studies from our laboratory have demonstrated that hGH is frequently expressed in mammary and endometrial carcinomas, associated with dissemination and is positively correlated with worse clinical outcomes in patients with these carcinomas [21]. Moreover, increased expression of hGH has been reported in endometriosis and endometrial adenocarcinoma compared to the normal uterine epithelium [22]. Furthermore, autocrine hGH has been shown to stimulate cell proliferation and survival of mammary carcinoma cells [23, 24], promote oncogenicity of mammary epithelial cells and tumor formation *in vivo* [25]. Autocrine hGH has also been demonstrated to promote EMT characterized by altered cell morphology, increased cell migration and invasion, as well as the increased mesenchymal and decreased epithelial markers expression in both mammary and endometrial carcinoma cells [26, 27]. The autocrine hGH-mediated EMT in breast cancer has been shown to be dependent on the hGH-stimulated increase in the expression of microRNA 96-182-183 cluster, which in turn suppressed breast cancer metastasis suppressor 1-like (BRMS1L) expression [28]. We have further shown that autocrine hGH enhanced the CSC-like properties, tumor initiating capacity, and invasive and metastatic capabilities of estrogen receptor negative (ER-) mammary carcinoma cells, suggestive of a critical role of autocrine hGH in tumor initiation and metastasis [29]. Additionally, autocrine hGH has been demonstrated to decrease the sensitivity of breast and endometrial cells towards ionising radiation (IR)-based therapy [30]. Recently, we have also reported that hGH expression is increased in hepatocellular carcinoma (HCC) as compared to normal liver specimens, with higher hGH expression being associated with higher tumor size, tumor grade and worse survival outcomes in HCC patients [31]. Similarly, we have demonstrated that autocrine hGH stimulated HCC progression by enhancing oncogenicity and tumor growth [31]. In addition, the functional roles of the hGH/hGHR signaling axis in melanoma, pancreatic cancer, glioma and craniopharyngioma have also been reported [32–37].

Previous studies have reported that the expression of growth hormone receptor (GHR) is increased in CRC compared to the normal mucosal tissue, and is positively associated with tumor size, tumor differentiation and pathological stage [38, 39], suggestive of the potential oncogenic role of either endocrine or tumor-derived hGH in CRC progression. More recently, it has been demonstrated that pituitary-derived hGH predisposes to the development of CRC, that was circumvented by the inhibition of hGHR signaling [40]. The same study has also reported increased localized expression of hGH in the stromal cells of colonic carcinoma [40]. However, the specific functional role of tumor derived hGH in CRC progression remains largely to be determined.

Herein, we demonstrated that elevated hGH expression is more frequently observed in CRC as compared to normal colorectal tissues, and is positively correlated with tumor size and lymph node metastasis. Additionally, hGH stimulated oncogenicity and EMT in CRC cells via the ERK1/2 signaling pathway and enhanced CSC-like behavior in an E-CADHERIN-dependent manner. Furthermore, autocrine production of hGH in CRC cells resulted in stimulation of tumor growth and invasive phenotype *in vivo*. Hence, inhibition of hGH may be a potential novel therapeutic approach for treatment of colorectal cancer.

## RESULTS

### The expression of hGH in CRC is positively associated with tumor size and lymph node metastasis

**Figure 1:**
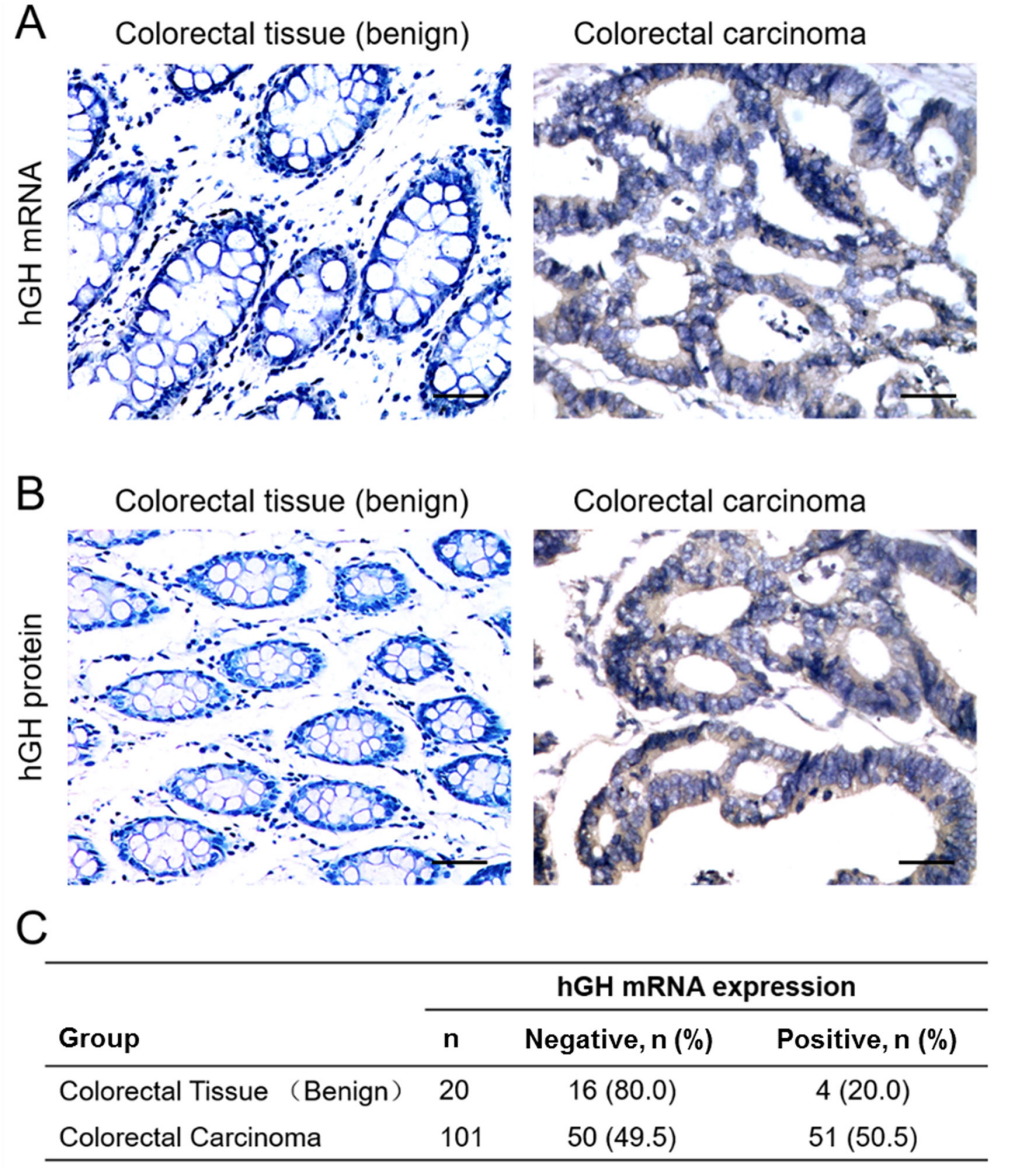
Expression of hGH in benign colorectal tissue and colorectal carcinoma (CRC) **(A)** *In situ* hybridization analysis of *hGH* mRNA expression in normal colorectal normal tissue and CRC. Images were counterstained with hematoxylin and captured at ×400 magnification. **(B)** Immunohistochemical analysis of hGH protein expression in normal colorectal tissue and CRC. Images were counterstained with hematoxylin and captured at ×200 magnification. Positive reactivity to hGH mRNA or protein is indicated by the brown color. **(C)** Percentages of normal colorectal tissue and CRC positive for *hGH* mRNA (p<0.05).

The expression of hGH mRNA and protein were examined by *in situ* hybridization (ISH) and immunohistochemistry (IHC) in both normal colorectal tissue and CRC respectively. Increased hGH mRNA and protein expression were observed in CRC, as compared to normal colorectal tissue (Figure 1A and 1B). Statistical analysis of *hGH* mRNA expression in 101 CRC and 20 normal colorectal tissue specimens revealed that a significantly higher percentage of CRC specimens (50.5%) were positive for *hGH* mRNA as compared to 20% in normal colorectal tissues from patients with benign disease (*P* = 0.012) (Figure 1C). Hence, *hGH* mRNA was more frequently expressed in CRC compared to benign colorectal tissue.

We further investigated the correlation of hGH expression with the clinicopathological features of CRC. As shown in Table 1, *hGH* mRNA expression was positively correlated with tumor size (*P* = 0.001) and lymph node metastasis (*P* = 0.003). However, no statistically significant correlation was observed between *hGH* mRNA expression and patient age, tumor grade or tumor stage.

**Table 1:** Correlation of *hGH* mRNA expression with clinicopathological parameters of CRC patients

Parameter	*n*	*hGH* positive expression, *n* (%)	*p* value
Age (years)			
≤ 55	40	16 (40.0)	0.088
> 55	61	35 (57.4)	
Tumor size (cm)			
≤ 5	60	22 (36.7)	**0.001**
> 5	41	29 (70.7)	
Lymph node metastasis			
Absent	38	12 (31.6)	**0.003**
Present	63	39 (61.9)	
Grade			
Well	8	4 (50.0)	0.397
Moderate	50	22 (44.0)	
Poor	43	25 (58.1)	
Stage			
I + II	38	18 (47.4)	0.625
III+ IV	63	33 (52.4)	

### Forced expression of hGH promotes proliferation, survival and oncogenicity of CRC cells *in vitro* and xenograft growth *in vivo*

**Figure 2:**
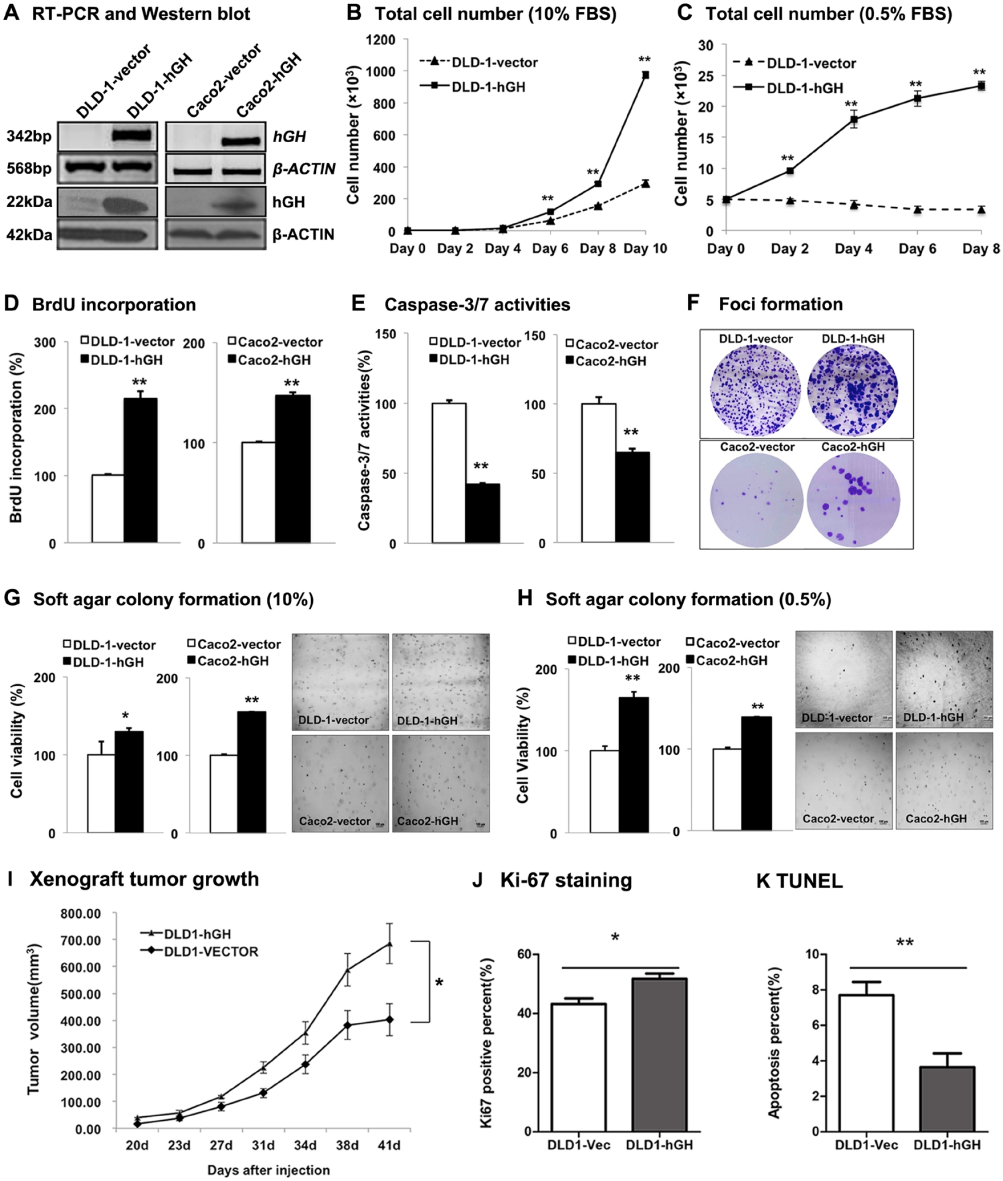
Forced expression of hGH stimulated cell proliferation, survival and oncogenicity in CRC cells, and promoted tumor growth *in vivo* DLD-1 and Caco2 cells were stably transfected with an expression vector containing *hGH* cDNA (designated as DLD-1-hGH and Caco2-hGH cells), or an empty vector as control (designated as DLD-1-vector and Caco2-vector cells). **(A)** Semi-quantitative RT-PCR and western blot analysis were used to examine hGH mRNA and protein levels respectively in stably transfected DLD-1 and Caco2 cells. β-ACTIN was used as input control. **(B)** Total cell number of DLD-1-vector and DLD-1-hGH cells over 10 days of culture in 10% FBS media, and **(C)** over 8 days of culture in 0.5% FBS media. **(D)** BrdU incorporation assay was performed to examine cell proliferation (S phase entry) in DLD-1-hGH and Caco2-hGH cells. Results were presented as percentages relative to the respective control cells. **(E)** Cell apoptosis was determined by measuring Caspase-3/7 activities in DLD-1-hGH and Caco2-hGH cells after serum starvation for 24 hours. Results were presented as percentages relative to the respective control cells. **(F)** Foci formation of DLD-1 and Caco2 stable cells was examined by crystal violet staining after two weeks of culture in media supplemented with 10% FBS. **(G)** Colony formation of DLD-1 and Caco2 stable cells in soft agar were examined after ten days of culture in 10% FBS media or **(H)** in 0.5% FBS media. Cell viability was measured by AlamarBlue assay. **(I)** Xenograft tumor growth generated by the subcutaneous injection of either DLD-1-vector or DLD-1-hGH cells into the subscapular region of nude mice. Tumors were formed 20 days following injection and tumor volumes were measured every 3-4 days. **(J)** Cell proliferation was examined by Ki-67 staining on the tumor sections. **(K)** Cell apoptosis in xenograft tumors was examined by TUNEL labeling. Images were captured under 400 × magnification. bp, base pair; kDa, kiloDalton. ^*^, p<0.05; ^**^, p<0.01.

To study the functional effect of hGH on CRC progression, we stably transfected two CRC cell lines, DLD-1 and Caco2, with an expression vector containing the full length *hGH* cDNA (designated DLD-1-hGH and Caco2-hGH cells respectively) or an empty vector as control (designated DLD-1-vector and Caco2-vector cells respectively). As demonstrated by semi-quantitative RT-PCR and western blot analysis, stable transfection of the hGH expression plasmid in CRC cells resulted in increased expression of hGH mRNA and protein, respectively (Figure 2A).

In monolayer culture, total cell number of DLD-1-hGH cells increased significantly more than DLD-1-vector cells over 10 days of culture in full serum (10% FBS) and reduced serum (0.5% FBS) media (Figure 2B and 2C). Moreover, forced expression of hGH significantly increased cell cycle S phase entry as shown by increased BrdU incorporation in both DLD-1-hGH and Caco2-hGH cells as compared to their respective control cells (Figure 2D). Furthermore, DLD-1-hGH and Caco2-hGH cells exhibited significantly decreased apoptosis as indicated by reduction in caspase-3/7 activities as compared to their respective control cells (Figure 2E). Thus, forced expression hGH in CRC cells increased total cell number through increasing cell cycle progression and decreasing apoptosis.

Anchorage-independent cell growth has been considered as one of the characteristics of oncogenically transformed cells [41]. Thus, we next examined the effect of forced expression of hGH on anchorage-independent growth in CRC cells using foci formation and soft agar colony formation assays. Enhanced foci formation was observed in DLD-1-hGH and Caco2-hGH cells compared to their respective control cells (Figure 2F). Concordantly, DLD-1-hGH and Caco2-hGH cells exhibited significantly increased capacity for colony formation in soft agar compared to DLD-1-vector and Caco2-vector cells respectively in both full serum and reduced serum (0.5% FBS) conditions (Figure 2G and 2H). These observations indicate that forced expression of hGH enhanced the anchorage-independent growth capacity of CRC cells.

To further determine the functional potential of autocrine hGH *in vivo*, we subcutaneously injected either DLD-1-vector or DLD-1-hGH cells into the subscapular region of nude mice. Both of the cell lines formed palpable and measurable tumors 20 days after injection. Tumors formed by DLD-1-hGH cells grew faster and exhibited a significantly greater tumor volume after 41 days, compared to the tumors formed by DLD-1-vector cells (Figure 2I). In addition, Ki-67 staining and TUNEL assay were performed to determine cell proliferation and apoptosis respectively in the tumor sections respectively [42, 43]. DLD-1-hGH tumors exhibited a significantly higher percentage of Ki-67 positive cells and a higher intensity of Ki-67 staining compared to DLD-1-vector tumors (Figure 2J). In contrast, DLD-1-hGH tumors displayed a lower percentage of TUNEL-labeled cells than the tumors generated by DLD-1-vector cells (Figure 2K). Hence autocrine hGH promotes tumor growth by increasing CRC cell proliferation and survival *in vivo*.

### Forced expression of hGH stimulates epithelial to mesenchymal transition in CRC cells *in vitro* and promotes local invasion *in vivo*

**Figure 3:**
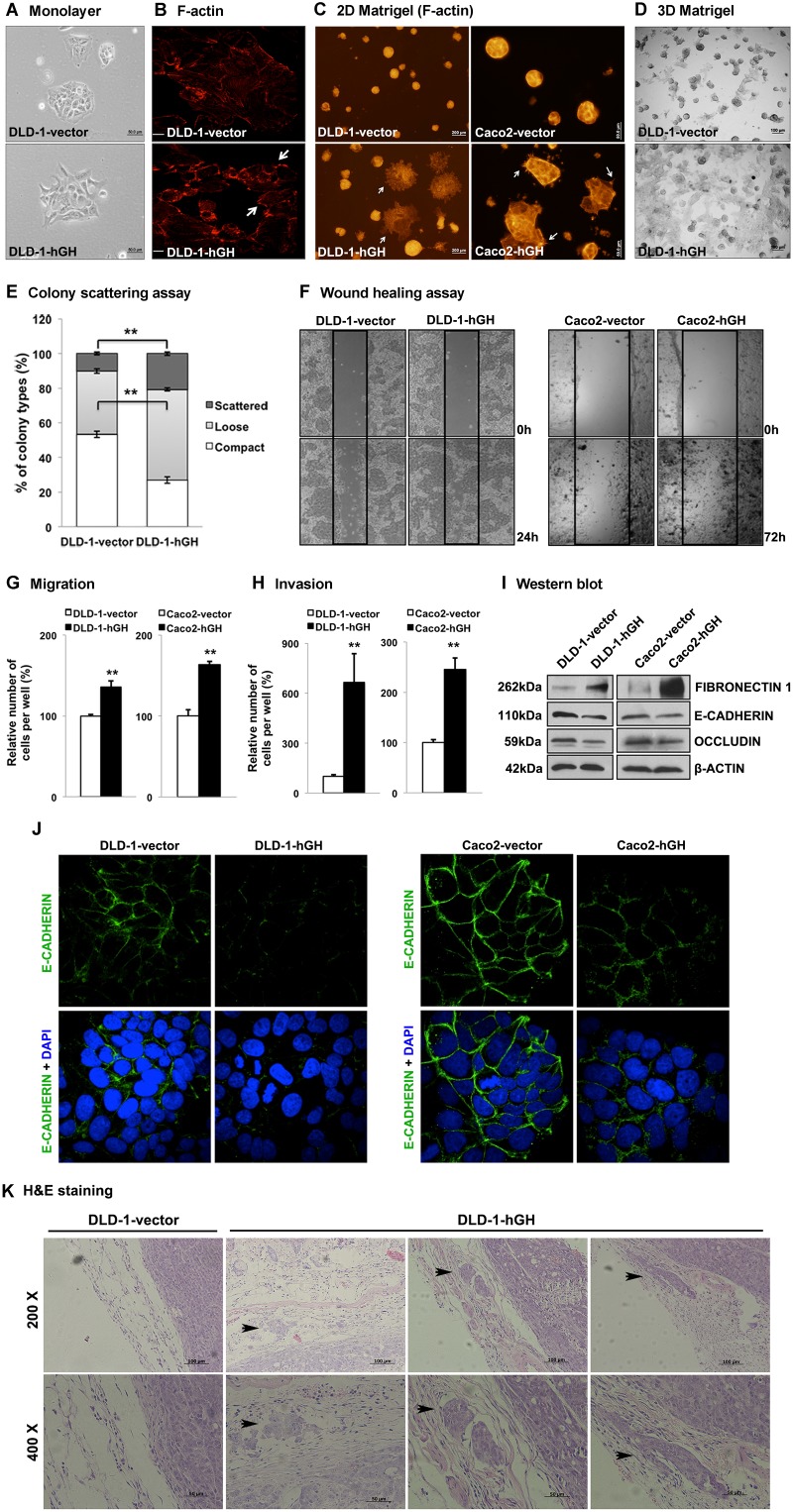
Forced expression of hGH stimulated epithelial to mesenchymal transition (EMT) in CRC cells and promoted local invasion *in vivo* **(A)** DLD-1-vector and -hGH cells were grown in monolayer adherent culture to approximately 40% confluence and the morphology of the cells were imaged at 200 × magnification. Bar, 50μm. **(B)** DLD-1-vector and -hGH cells were stained for F-actin with rhodamine-phalloidin (red) and images were obtained with a confocal fluorescence microscope. The lamellipodial protrusions (*arrows*) at the leading edges of the cells are indicated. Bar, 20μm. **(C)** DLD-1 and Caco2 stable cells were grown on 2D Matrigel and stained for F-actin. DLD-1-hGH and Caca2-hGH cells exhibited a distinct morphology with numerous protrusions on the edge of the colonies (arrows), which were absent in the respective vector cells. Bar, 200μm (DLD-1), 50μm (Caco2). **(D)** DLD-1 cells were grown in 3D Matrigel. Images were captured under 40 × magnification. Bar, 100μm. **(E)** Colony scattering assay. DLD-1-vector and -hGH cells were seeded at a very low density, and colonies formed by these cells were categorized as scattered, loose or compact. The percentages of colonies in each category are presented. **(F)** Cell migration of DLD-1 and Caco2 stable cells were examined using the wound healing assay. Wounded areas (between the straight lines) were imaged under 100 × magnification. **(G)** Cell migration and **(H)** invasion of DLD-1-hGH and Caco2-hGH cells were examined using transwell assays. After 48 hours, cells that have migrated or invaded through the transwell membrane were stained with Hoechst 33342 and counted under a fluorescence microscope. Results were presented as percentages relative to the respective control cells. **(I)** The protein expression of epithelial and mesenchymal markers in DLD-1 and Caco2 stable cells were analysed using western blot. β-ACTIN was used as an input control. The sizes of detected protein bands are shown on the left side. **(J)** DLD-1 and Caco2 stable cells were fixed and stained with E-cadherin antibodies, followed by fluorescent dye-tagged secondary antibodies. DAPI was used to stain cell nuclei. E-cadherin (green), and DAPI (blue) were visualized by confocal fluorescence microscopy under 1000 × magnification. **(K)** The tumors and adjacent tissues were stained with hematoxylin and eosin. The local invasion of DLD-1-hGH tumors into adjacent tissues are indicated (*black arrows*). ^**^, p<0.01.

During cancer progression, increased invasiveness of carcinoma cells is commonly accompanied by phenotypic conversion including changes in cell morphology, cell motility and gene expression [9]. In monolayer adherent culture, DLD-1-hGH cells exhibited elongated and irregular cell shape with loss of intercellular contact, appearing as a mesenchymal-like phenotype, whereas DLD-1-vector cells were of polygonal shape with regular dimensions and grew in colonies with tight intercellular contact (Figure 3A), similar to their parental DLD-1 wild type cells. In addition, forced expression of hGH altered the organization of the actin cytoskeleton, which is associated with cell morphology, cell migration and adhesive properties [44], in DLD-1 cells. DLD-1-hGH cells displayed accumulated filamentous actin (F-actin) at the cell periphery, loss of well-organized stress fibers in the cytoplasm and formed multiple lamellipodial protrusions at the leading edges, whereas DLD-1-vector cells exhibited well-organized stress fibers near the cell periphery, as well as in the cytoplasm, and with a regular appearance of stress fibers (Figure 3B). The reorganization of the actin cytoskeleton that leads to the formation of lamellipodial protrusions provide the driving force for cell migration [45], suggesting a potential role of autocrine hGH in cell motility in CRC cells. Furthermore, colonies formed by DLD-1-hGH cells on 2D Matrigel exhibited a stellate organization with numerous protrusions formed by the cells extending and migrating from the main colony bulk, whereas DLD-1-vector cells grew as spherical colonies (Figure 3C). Similarly, colonies formed by Caco2-vector cells on 2D Matrigel exhibited a rounded and regular organization, whereas the colonies formed by Caco2-hGH cells exhibited an irregular shape with several protrusions on the edge of colonies (Figure 3C). Three-dimensional (3D) culture in basement membrane matrix is widely utilized to simulate the *in vivo* cell behavior *in vitro*, including cell invasion [46]. DLD-1-vector cells in 3D culture mostly generated spheroidal colonies, whereas most of the colonies generated by DLD-1-hGH cells displayed an irregular organization with multiple protrusions (Figure 3D). In the colony scattering assay, DLD-1-hGH cells generated a significantly larger proportion (2-fold increase) of scattered colonies and a smaller proportion (2-fold decrease) of compact colonies, compared to DLD-1-vector cells (Figure 3E). Additionally, DLD-1-hGH and Caco2-hGH cells exhibited significantly increased cell migration in wound healing assays and transwell migration assays, as well as cell invasion in transwell invasion assays, compared to their respective control cells (Figure 3F, 3G and 3H).

Altered cell morphology, and increased cell migration and invasion observed with forced expression of hGH suggests that autocrine hGH is involved in stimulating EMT in CRC cells. Quantitative-PCR (qPCR) analysis demonstrated that forced expression of hGH in DLD-1 and Caco2 cells significantly decreased the mRNA levels of epithelial marker genes, *CDH1* and *OCLN*, increased the mRNA levels of mesenchymal marker genes, *VIM*, *FN1*, *CHD2* and *IGF-1*, and also promoted mRNA expression of the metastatic marker gene *MMP9*, compared to their respective control cells (Table 2). The protein levels of epithelial markers E-CADHERIN and OCCLUDIN were significantly decreased, whereas that of the mesenchymal marker FIBRONECTIN 1 was significantly increased, in DLD-1 and Caco2 cells with forced expression of hGH (Figure 3I). Furthermore, forced expression of hGH in DLD-1 and Caco2 cells resulted in decreased cell membrane and cellular contact localization of E-CADHERIN (Figure 3J). Hence, autocrine hGH stimulates EMT in CRC cells through the promotion of the classical EMT gene expression pattern.

**Table 2:** qPCR analysis of the effect of forced expression of hGH on expression of genes involved in EMT and metastatic progression of CRC cells

		DLD-1		Caco2	
**Gene function**	**Gene**	**Fold change**	**p-value**	**Fold change**	**p-value**
**Epithelial**	*CDH1*	0.12	9.18E-05	0.10	0.0285
	*OCLN*	0.26	1.84E-04	0.16	0.0118
	*CTNNA1*	1.54	2.58E-04	0.73	0.0062
	*CTNNB1*	0.94	6.21E-03	0.80	0.0218
	*CTNND1*	2.17	3.55E-03	0.37	0.0286
**Mesenchymal**	*TWIST1*	1.15	2.72E-03	5.71	0.0043
	*VIM*	8.5	1.09E-03	3.44	0.0216
	*FN1*	6.58	1.47E-03	10.55	0.0260
	*CDH2*	5.22	5.33E-03	28.18	0.0108
	*SNAI1*	3.85	9.57E-04	1.52	0.2038
	*SNAI2*	1.15	1.06E-01	2.57	0.3227
	*IGF-1*	12.42	1.96E-03	6.49	0.0162
	*ZEB1*	1.27	2.60E-02	2.00	0.4663
	*ZEB2*	1.13	3.88E-02	1.84	0.6589
**Metastatic**	*MET*	1.69	8.65E-05	4.33	0.0297
	*MMP2*	1	4.46E-01	4.01	0.0359
	*MMP9*	7.72	5.24E-03	3.61	0.1914
	*MTA1*	1.97	5.19E-05	6.70	0.1663
	*MTA2*	2.21	5.53E-04	0.88	0.1341
	*NME1*	2.24	2.48E-04	2.55	0.1546
	*PLAU*	0.82	4.73E-02	7.16	0.0163
	*PLAUR*	0.91	3.32E-02	20.49	0.0080

Results are represented as fold change in mRNA levels in DLD-1-hGH or Caco2-hGH cells relative to the respective control cells.

To further determine whether autocrine hGH promotes local invasion of CRC cells *in vivo*, we carried out hematocylin/eosin (H&E) staining on xenograft tumors together with the adjacent tissues. The tumors generated from DLD-1-vector cells exhibited a well-defined boundary without any infiltration into the surrounding normal tissues, whereas the tumors generated from DLD-1-hGH cells infiltrated into the surrounding tissues to form additional tumor islands (Figure 3K).

### Inhibition of ERK1/2 activity abrogates hGH-stimulated oncogenicity and EMT in CRC cells

**Figure 4:**
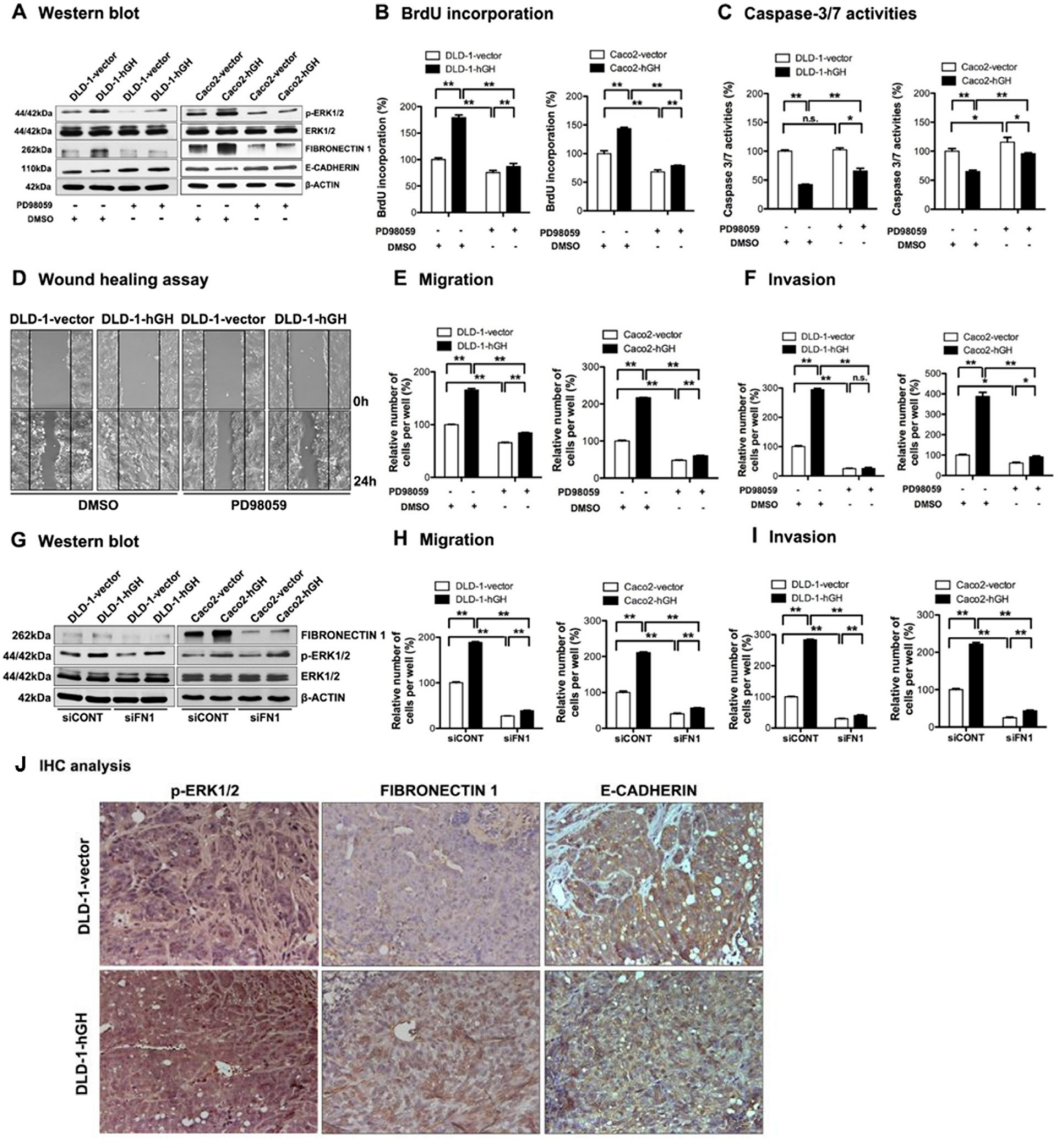
Inhibition of ERK1/2 activity significantly abrogated hGH-stimulated oncogenicity and EMT in CRC cells **(A-F)** DLD-1 and Caco2-vector and –hGH cells were treated with either DMSO vehicle or PD98059 (20μM) for 24 hours. (A) The levels of ERK1/2 and p-ERK1/2, as well as EMT markers FIBRONECTIN 1 and E-CADHERIN, were examined by western blot analysis. β-ACTIN was used as input control. (B) Cell proliferation of DLD-1 and Caco2 stable cells ± PD98059 (20μM) was evaluated with BrdU incorporation assay. Results are presented as percentages relative to the untreated vector cells. (C) Cell apoptosis of DLD-1 and Caco2 stable cells ± PD98059 (20μM) was evaluated with Caspase-3/7 assay. Results are presented as percentages relative to the untreated vector cells. (D) Wound healing assay was performed in DLD-1-vector and DLD-1-hGH cells ± PD98059 (20μM). (E) Cell migration and (F) cell invasion of DLD-1 and Caco2 stable cells ± PD98059 (20μM) were evaluated using Transwell assays. **(G-I)** DLD-1 and Caco2-vector and –hGH cells were transiently transfected with either scrambled siRNA (siCONT) or siRNA against FIBRONECTIN 1 (siFN1). (G) The levels of ERK1/2 and p-ERK1/2, and mesenchymal marker FIBRONECTIN 1, in DLD-1 and Caco2 stable cells with siRNA-mediated depletion of FIBRONECTIN 1, were examined by western blot analysis. β-ACTIN was used as an input control. (H) Cell migration and (I) invasion of DLD-1 and Caco2 stable cells with siRNA-mediated depletion of FIBRONECTIN 1 were determined using transwell assays. **(J)** The phosphorylation levels of ERK1/2 and the expression levels of FIBRONECTIN 1 and E-CADHERIN in primary tumors were examined by IHC analysis after subcutaneous injection. Images were captured under 400 × magnification. ^*^, p<0.05; ^**^, p<0.01; n.s., not significant.

It has previously been reported that the MAPK/ERK pathway plays an important role in hGH-mediated cell functions [47]. In order to determine the potential mechanisms involved in autocrine hGH-mediated oncogenicity, we performed western blot analysis to examine the phosphorylation status of ERK1/2 in the –hGH and –vector CRC cells. Increased levels of phosphorylated ERK1/2 (p-ERK1/2) were observed in DLD-1 and Caco2 cells with forced expression of hGH compared to their respective control cells, whereas the expression of total ERK1/2 remained unchanged (Figure 4A). To determine if hGH-mediated oncogenicity is ERK1/2-dependent, we used a MEK inhibitor, PD98059, to specifically inhibit MEK phosphorylation of ERK1/2 [48]. Treatment with PD98059 significantly reduced the levels of p-ERK1/2 in DLD-1 and Caco2 stable cells, but did not affect the levels of total ERK1/2 (Figure 4A). Moreover, PD98059 inhibition of ERK1/2 significantly decreased BrdU incorporation and increased Caspase-3/7 activities in both DLD-1 and Caco2 –vector and –hGH cells (Figure 4B and 4C). Notably, PD98059 treatment abrogated the autocrine hGH-mediated increase in BrdU incorporation and decrease in Caspase-3/7 activities in DLD-1-hGH and Caco2-hGH cells to the levels of their respective control cells (Figure 4B and 4C). Thus, autocrine hGH utilizes the ERK1/2 pathway to promote cell proliferation and survival in CRC cells.

In addition, the inhibition of ERK1/2 by PD98059 significantly decreased CRC cell migration in both wound healing and transwell migration assays (Figure 4D and 4E), and decreased cell invasion in the transwell invasion assay (Figure 4F). In particular, PD98059 treatment abrogated the autocrine hGH-stimulated increase in cell migration and invasion in DLD-1-hGH and Caco2-hGH cells to levels similar to or lower than that of the control vector cells (Figure 4D, 4E and 4F). As we observed that autocrine hGH acts through the ERK1/2 pathway to promote migration and invasion of CRC cells, we then determined whether the hGH-mediated activation of ERK1/2 modulates the expression of EMT-related genes. DLD-1-hGH and Caco2-hGH cells exhibited significantly increased FIBRONECTIN 1 (Supplementary Figure 1A and Figure 4A) and decreased E-CADHERIN (Supplementary Figure 1B and Figure 4A) promoter activities and protein levels, compared to their respective control cells. The inhibition of ERK1/2 by PD98059 significantly decreased FIBRONECTIN-1 and increased E-CADHERIN expression in these cells (Supplementary Figure 1 and Figure 4A). Notably, PD98059 treatment abrogated the hGH-mediated upregulation of FIBRONECTIN 1 expression and downregulation of E-CADHERIN expression in DLD-1-hGH and Caco2-hGH cells (Supplementary Figure 1 and Figure 4A). Next, we determined whether hGH acts through the ERK1/2 pathway to modulate FIBRONECTIN 1 and E-CADHERIN expression, thereby effecting EMT in the CRC cells. The depletion of FIBRONECTIN 1 in DLD-1 and Caco2 cells did not affect the activation of ERK1/2 *i.e.* pERK1/2 levels (Figure 4G), although FIBRONECTIN-1 depletion significantly abrogated the hGH-stimulated increase in cell migration and invasion (Figure 4H and 4I). Also, forced expression of E-CADHERIN in DLD-1 cells did not affect the activation of ERK1/2 *i.e.* pERK1/2 levels (Supplementary Figure 2A), but abrogated the hGH-stimulated increase in cell migration and invasion (Supplementary Figure 2B and 2C). Therefore, autocrine hGH expression in CRC cells stimulated EMT via the ERK1/2 pathway, dependent on the downstream modulation of FIBRONECTIN 1 and E-CADHERIN expression.

Furthermore, we performed IHC analysis to examine the levels of p-ERK1/2, and EMT markers FIBRONECTIN 1 and E-CADHERIN in the xenograft tumor sections. Increased nuclear accumulation of p-ERK1/2 was observed in DLD-1-hGH tumors compared with DLD-1-vector tumors (Figure 4J). Additionally, DLD-1-hGH tumors exhibited higher protein levels of FIBRONECTIN 1 and lower protein levels of E-CADHERIN compared to DLD-1-vector tumors (Figure 4J). Consistent with our *in vitro* findings, autocrine hGH expression in CRC cells promotes tumor growth and EMT *in vivo*, through the activation of the ERK1/2 pathway and modulation of EMT markers.

### Forced expression of hGH stimulates CSC-like behavior in CRC cells in an E-CADHERIN-dependent manner

**Figure 5:**
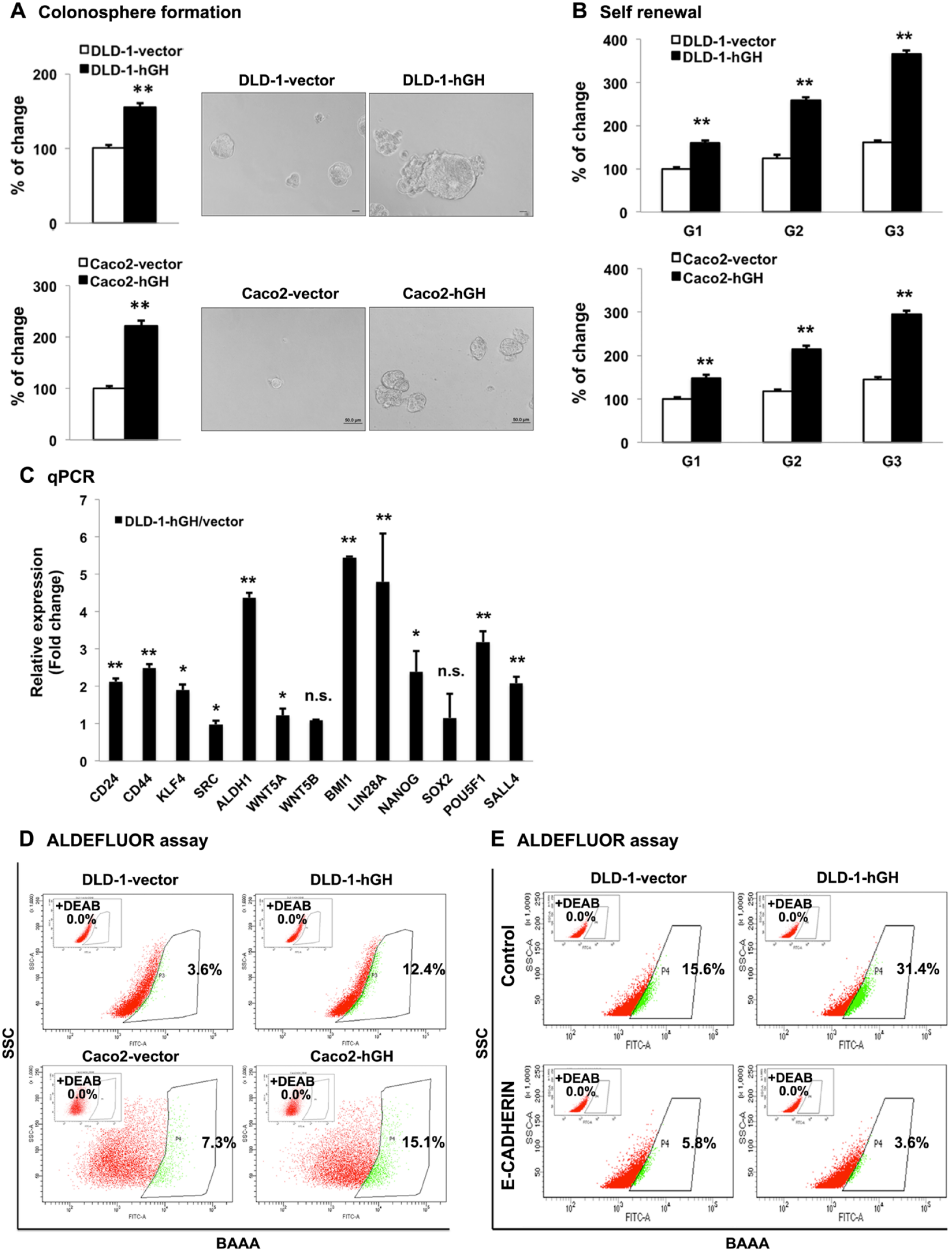
Forced expression of hGH promoted CSC-like behavior in CRC cells in a manner dependent on E-CADHERIN repression **(A)** DLD-1 and Caco2 –vector and –hGH cells were seeded in ultra low attachment plates in colonosphere culture media. The numbers of the colonospheres were counted after 7 days (for DLD-1 cells) or 10 days (for Caco2 cells). Images of colonospheres were captured under 200 × magnification. Bar, 20μm (DLD-1 cells); 50μm (Caco2 cells). Results were presented as percentages relative to the respective control cells. **(B)** Colonosphere formation of DLD-1 and Caco2 stable cells from the first generation (G1) to third generation (G3). The numbers of colonospheres in each generation were counted after 7 days (for DLD-1 cells) or 10 days (for Caco2 cells). Results were presented as percentages relative to the respective G1 vector cells. **(C)** The mRNA levels of CSC markers in DLD-1-vector and –hGH cells were analyzed by qPCR. *β-ACTIN* was used as input control. Results are presented as fold change in mRNA levels in DLD-1-hGH cells relative to DLD-1-vector cells. **(D)** The ALDH1+ population was determined in DLD-1 and Caco2 stable cells using the ALDEFLUOR assay. Cells were incubated with Aldefluor substrate (BAAA, BODIPY®-aminoacetaldehyde) to define the ALDH1 positive population, and a specific inhibitor of ALDH1, diethylaminobenzaldehyde (DEAB), was used as a control to establish the baseline fluorescence. Flow cytometry analysis was used to indicate side scatter (SSC) and fluorescence intensity. **(E)** The ALDH1+ cell population was determined in DLD-1 stable cells with forced expression of E-CADHERIN. ^*^, p<0.05; ^**^, p<0.01; n.s., not significant.

Recent studies have reported that EMT and CSC-like behavior are closely linked [49, 50]. As we observed that forced expression of hGH stimulates EMT, we further examined the potential function of autocrine hGH in the acquisition of CSC-like behavior in CRC cells. Forced expression of hGH significantly promoted colonosphere formation of CRC cells, with an increased number of colonospheres formed by DLD-1-hGH and Caco2-hGH cells compared with the respective vector cells (Figure 5A). Furthermore, the colonospheres formed by DLD-1-hGH and Caco2-hGH cells were observed to be larger in size compared with those formed by the respective control cells (Figure 5A). To further confirm that autocrine hGH-promoted colonosphere formation arose from the self-renewal of individual cells rather than cell aggregation, we performed a self-renewal assay to examine the colonosphere growth of DLD-1 and Caco2 stable cells over three generations [51]. DLD-1 and Caco2 cells with forced expression of hGH exhibited significantly increased numbers of secondary and tertiary colonospheres, compared to their respective control cells (Figure 5B). In addition, increased mRNA levels of CSC marker genes, *CD24*, *CD44*, *KLF4*, *ALDH1*, *BMI1*, *LIN28A*, *NANOG*, *POU5F1* and *SALL4* were observed in DLD-1-hGH cells compared to DLD-1-vector cells (Figure 5C). The aldehyde dehydrogenase I (ALDH1) has been identified as one of the markers for CSCs in cancer including colorectal cancer [52]. In the ALDEFLUOR assay, forced expression of hGH in DLD-1 and Caco2 cells significantly increased the percentage of ALDH1^+^ cells by 3.44-fold and 2.07-fold respectively, compared to their corresponding vector cells (Figure 5D). Collectively, these results suggest that hGH increases CSC marker gene expression and promotes CSC-like behavior in CRC cells.

It has previously been reported that the loss of membranous E-CADHERIN is essential for Wnt/β-catenin promotion of the cancer stem cell phenotype [40]. As we demonstrated that loss of E-CADHERIN was essential for hGH-stimulated EMT in CRC cells, we next examined the functional role of E-CADHERIN in the autocrine hGH-stimulated increase in CSC-like behavior in CRC cells. Forced expression of E-CADHERIN in DLD-1-vector and DLD-1-hGH cells significantly decreased the ALDH1^+^ cell population, and abrogated the autocrine hGH-stimulated increase in the ALDH1^+^ cell population of DLD-1-hGH cells (Figure 5E). These observations suggest that the loss of E-CADHERIN is required for the autocrine hGH-stimulated increase in ALDH1^+^ CSC population. Thus, forced expression of hGH in CRC cells promotes CSC-like behavior dependent on E-cadherin repression.

## DISCUSSION

Colorectal cancer remains a major health burden worldwide, especially advanced colorectal malignancy that tends to poorly respond poorly to current therapeutic strategies [53]. It is essential to understand the biological events that contribute to CRC progression, that result in invasive and eventually metastatic malignancies. Recent evidence has demonstrated the critical role of hGH in the development of mammary, endometrial, hepatocellular and pancreatic carcinomas, and melanoma [21, 31, 32, 36, 54]. Herein, we demonstrated the significant role of hGH in the acquisition of oncogenic and invasive properties of CRC cells, including its function in promoting oncogenicity, EMT and CSC-like behavior.

In this study, we observed autocrine expression of hGH in normal colorectal tissue and CRC, with CRC exhibiting increased hGH expression. Furthermore, the *hGH* mRNA expression level in CRC was associated with larger tumor size and lymph node metastasis. A similar association of tumor hGH expression and worse clinical outcomes has been demonstrated in mammary, endometrial and hepatocellular carcinomas [21, 31]. Autocrine hGH expression has been demonstrated to be positively associated with tumor stage and lymph nodes metastasis in mammary carcinoma, and positively associated with FIGO grade, myometrial invasion and ovarian metastasis in endometrial carcinoma [21]. Furthermore, autocrine hGH expression has been correlated with increased tumor size and stage, and poorer relapse-free and overall survival in HCC patients [31]. These clinical findings suggest an oncogenic role of locally produced hGH in cancer progression, including CRC.

Herein, we showed that hGH enhanced the cell proliferation, survival and oncogenicity of CRC cells, both *in vitro* and in xenograft model *in vivo*. This oncogenic effect of autocrine hGH has previously been reported in other cancers [23–25, 27, 31]. Autocrine hGH has been demonstrated to stimulate the oncogenic transformation of immortalized human mammary epithelial cells [25], increase mitogenesis and reduce apoptotic cell death of mammary carcinoma cells [23, 24], and promote mammary tumor growth *in vivo* [25], which strongly support the oncogenic role of hGH in mammary carcinomas. Similarly, autocrine hGH has been shown to enhance cell proliferation and survival in endometrial carcinoma cells, and promote tumor growth in xenograft mice [27]. Consistently, autocrine hGH has also been demonstrated to stimulate the oncogenicity and tumor growth of HCC cells *in vitro* and *in vivo* [31]. In addition, hGH signaling has been reported to increase the survival and proliferation of pancreatic ductal adenocarcinoma (PDAC) and melanoma cells [32, 36].

Chesnokova *et al.*, has reported that excess pituitary-derived growth hormone (GH) predisposes to the development of precancerous colon polyps and colon cancer, while inactivating growth hormone receptor (GHR) mutations or GHR antagonist attenuates the development of colon cancer [40]. These observations support the role of endocrine hGH in the development of colon cancer. In addition, the levels of GHR, which is expressed in both colonic epithelial and stromal cells, was also found to be increased in epithelial colon adenocarcinoma cells as compared to normal colon tissue [40]. Similarly, upregulated GHR expression in CRC has been previously reported [21]. Along with our current study, these findings suggest the involvement of local hGH signaling in the development of CRC. We observed increased hGH expression in the epithelial cells in CRC at both the mRNA and protein levels, indicating that the CRC cells are the sites of synthesis of hGH. The localization of hGH protein in the varying cell types (stromal) in the study by Chesnokova *et al.* [40], may not represent the sites of hGH synthesis but the uptake of hGH as a secreted protein [21], likely accounting for the discrepancy in the localization of hGH protein. In line with our study, both hGHR and hGH proteins have previously been reported to be frequently expressed in CRC [55]. Furthermore, our previous studies have found that hGH expression is mainly localized to carcinoma cells in several other cancers including mammary, endometrial and hepatocellular carcinomas [21, 31]. Moreover, hGH expression has also been observed in a variety of carcinoma cell lines such as human mammary carcinoma cells [29, 56], endometrial carcinoma cells [27], hepatocellular carcinoma cells [31], and herein at low levels in CRC cells. We have further validated that forced expression of hGH in CRC cells functionally promoted cell proliferation and survival, oncogenicity, EMT and CSC-like properties, acting in autocrine and paracrine manner. Presumably, paracrine hGH from stromal cells would exert similar functional effects on CRC cells as reported by Chesnokova *et al.* [40]. In accordance with our study, the autocrine actions of hGH in promoting oncogenic behaviour in several cancers, including mammary, endometrial and hepatocellular carcinomas, have been reported [21, 23, 27, 31, 57].

EMT is a transitional process, in which epithelial cells acquire the mesenchymal characteristics and convert into mesenchymal-like cells [6]. During cancer progression, EMT is thought to contribute to metastasis and enhanced cell invasiveness [58]. Herein, we demonstrated that autocrine hGH stimulated EMT with morphologic conversion, enhanced cell migratory and invasive properties *in vitro*, as well as local invasion *in vivo*. These observations were consistent with previous findings that autocrine production of hGH promoted cell migration and invasion in mammary and endometrial carcinoma cells [26, 27, 29]. One mechanism through which autocrine hGH acts to promote EMT has been reported to be the hGH-GHR-STAT3/STAT5-miR-96-182-183-BRMS1L-ZEB1/E47-EMT/invasion axis [28]. Similarly in melanoma and PDAC, the hGH-GHR signaling has been reported to enhance EMT and metastasis [32, 33, 36, 37].

Several pathways activated by hGH signaling, including the ERK1/2 pathway [20, 54], have been reported to contribute to increased oncogenic behavior and EMT [57, 59]. We have previously demonstrated that the MAPK pathway is essential for autocrine hGH stimulation of mammary carcinoma cell proliferation, although autocrine hGH has also been shown to activate STAT5 transcriptional activity [23]. Nevertheless, STAT5 has been reported to possess opposing roles in cancers, including its dual oncogenic and tumor suppressive activities in breast cancer [60]. STAT3 has been reported to mediate the oncogenic actions of hGH in endometrial carcinoma cells [61]. However, we observed that STAT3 activity was minimally affected by forced expression of hGH in CRC cells yet there was a prominent activation of ERK1/2. Correspondingly, we showed that the inhibition of ERK1/2 activity with PD98059 largely abrogated autocrine hGH-stimulated cell proliferation, survival, invasion and migration of CRC cells. The activating mutations of the Ras/Raf/MAPK pathway are frequently observed in CRC [62, 63]. The DLD-1 cells used in this study possessed the G^13^D mutation in KRAS, resulting in the constitutive activation of KRAS and the MAPK pathway, whereas the Caco2 cells possessed wildtype KRAS and BRAF proteins [64]. Nevertheless, in both DLD-1 and Caco2 cells, we observed that the forced expression of hGH increases the activation of ERK1/2 independently of the RAS or RAF activating mutations, pointing to the critical role of hGH in activating ERK1/2 in CRC.

In addition, we observed that the hGH-mediated ERK1/2 activation led to repression of E-cadherin expression and stimulation of FIBRONECTIN 1 expression, which was abrogated by PD98059 inhibition of ERK1/2. Conversely, forced expression of E-CADHERIN or knockdown of FIBRONECTIN 1 did not affect the hGH-mediated activation of ERK1/2 in CRC cells, suggesting that E-CADHERIN and FIBRONECTIN 1 function downstream of the hGH-ERK1/2 axis in the CRC cells. Consistently, it has been reported that MAPK/ERK repressed E-CADHERIN expression [65] through MAPK/ERK-stimulated expression of SNAIL, TWIST, ZEB1 or ZEB2 transcription factors, which are known transcriptional repressors of E-CADHERIN [6]. Furthermore, the activated KRAS(G^12^D), upstream of ERK1/2 has been shown to reduce E-cadherin expression, and in turn increase invasive and migratory properties of pancreatic cancer cells [66]. The loss of epithelial marker E-CADHERIN has been considered as a hallmark of EMT [6]. E-CADHERIN is expressed in the adherent junctions of epithelial cells and plays a key role in maintaining cell polarity and intercellular adhesion, thereby suppressing cell invasion [67]. It is well established that induction of EMT is accompanied by decreased expression of E-CADHERIN [68]. Clinically, the downregulated expression of E-cadherin has been associated with poor differentiation, increased progression and metastasis of tumors, and poorer prognosis in CRC patients [69–72]. It has been observed that the central parts of the primary colorectal tumors contain cells that exhibit high membranous E-CADHERIN levels with co-localized β-catenin, whereas the cells at the invasive front of mesenchymal-like tumors exhibit loss of the membranous E-CADHERIN with predominant nuclear β-catenin [73]. Furthermore, depletion of E-CADHERIN in lung cancer cells has also been demonstrated to result in induction of EMT and increase in cell invasion [67]. The loss of E-CADHERIN has been reported to stimulate the translocation of β-catenin and activate the Wnt/β-catenin pathway, which is a potential mechanism of E-cadherin loss-induced EMT [74]. Activated β-catenin in the nucleus regulates transcription of specific genes, such as *c-Myc*, which is functionally involved in EMT [75]. Interestingly, Hollestelle *et al.* has demonstrated that forced expression of E-CADHERIN in MDA-MB-231 cells, characterized by mesenchymal phenotype and loss of E-CADHERIN expression, fail to revert the cells into an epithelial morphology nor alter other EMT markers expression, while in SKBR3 cells, forced expression of E-CADHERIN reverted the cells into an epithelial morphology [76]. In our study, hGH-stimulated migration or invasion, being EMT-associated cell behaviors, was abrogated by the forced expression of E-CADHERIN in CRC cells. This observation highlighted that repression of E-CADHERIN was required for hGH-stimulated EMT in CRC cells. Additionally, we also demonstrated that autocrine hGH increased expression of mesenchymal marker FIBRONECTIN 1, which is also required for hGH-stimulated cell migration and invasion. FIBRONECTIN 1 is an extracellular matrix protein with a key role in modulating cell adhesion and stimulating cell migration [77]. It has been demonstrated to bind integrins, such as α_5_β_1_ integrin, thereby activating receptor tyrosine kinases (RTKs) to promote cell migration [78]. Decreased expression of E-CADHERIN and increased expression of FIBRONECTIN 1 are commonly observed together in EMT [92], however, the inverse relationship between E-CADHERIN and FIBRONECTIN 1 expression has not been addressed. As it has been demonstrated that FIBRONECTIN 1 expression is transcriptionally activated by β-catenin-TCF/LEF [79], this provides a possibility that the nuclear localization of β-catenin induced by the loss of E-CADHERIN [74], can result in the stimulation of TCF/LEF-mediated transcription of FIBRONECTIN 1. Therefore, one plausible mechanism that the hGH-ERK1/2-mediated decrease in E-CADHERIN expression may increase FIBRONECTIN 1 expression is through the activation of the Wnt pathway and nuclear localization of β-catenin.

Increasing evidence suggests that stem cells play a critical role in cancer progression besides in normal tissue development [80]. CSCs are also thought to contribute to the development of chemo-resistance and relapse following chemotherapy [81], which is a major problem responsible for over 90% of drug failure in metastatic cancers [82]. A recent study has revealed a functional role of hGH in stem and progenitor cells [83]. Lombardi *et al.* demonstrated that GHR is co-expressed with stem cell markers in a subpopulation of normal human mammary epithelial cells (HMECs), and these GHR positive cells exhibited increased CSC-like behavior as compared to GHR negative cells [83]. Additionally, the activation of GHR with hGH treatment was shown to enhance mammosphere formation in the GHR positive HMECs [83]. More recently, we have implicated autocrine hGH as a promoter of CSC-like behavior in ER-negative breast cancer cells, resulting in increased tumor initiation capacity [29]. In this study, we similarly demonstrated that autocrine hGH promoted the acquisition of CSC-like behavior in CRC cells, as characterized by increased colonosphere formation, self-renewal properties, ALDH1^+^ population and CSC markers expression. One potential mechanism of autocrine hGH in the promotion of CSC-like properties is its effect on EMT. Although the origin of CSCs is debatable, a number of studies suggest that they may arise by undergoing EMT [84]. The induction of EMT by overexpressing transcription factors SNAIL or TWIST in HMECs resulted in the acquisition of stem cell-like properties, while isolated stem cell-like cells from mammary glands or carcinomas exhibited expression of EMT markers [11]. Moreover, high expression of EMT markers has been reported to stimulate CSC-like behavior in breast, ovarian and colorectal carcinoma cells, and correlate with chemo- or radio-resistance and metastatic potential [85–87]. In this study, we observed that loss of E-CADHERIN was required for the hGH-stimulated increase in ALDH1^+^ cell population. The mechanism through which the loss of E-CADHERIN stimulates CSC properties has been reported to be potentially associated with the activation of the Wnt/β-catenin pathway [88].

In conclusion, hGH expression in CRC was positively associated with tumor size and lymph node metastasis. Forced expression of hGH in CRC cells stimulated oncogenicity and EMT via the ERK1/2 pathway. Furthermore, CSC-like behavior was co-induced with EMT by forced expression of hGH in CRC cells in an E-CADHERIN-dependent manner. These observations demonstrate a significant role of hGH in CRC progression and suggest that hGH may be considered as a potential therapeutic target to prevent CRC progression. As the autocrine hGH-stimulated oncogenic and EMT functions is at least partially ERK1/2 dependent, activating mutations in KRAS or BRAF would probably render the therapeutic inhibition of hGH less effective, while combination therapy with inhibitors of the MAPK pathway would potentially improve therapeutic efficacy in such cases.

## MATERIALS AND METHODS

### Cell culture and stable transfection

The human CRC cell lines, DLD-1 and Caco2, were purchased from American Type Culture Collection (ATCC, Manassas, VA), and cultured in ATCC recommended conditions. DLD-1 and Caco2 cells were stably transfected with an expression vector (pcDNA3.1) containing full length *hGH* gene (designated as DLD-1-hGH and Caco2-hGH cells) or the empty vector (designated as DLD-1-vector and Caco2-vector cells) as control by using FuGENE 6 Transfection Reagent (Promega, WI). Stably transfected cells were selected with complete media containing 1000μg/ml (for DLD-1 cells) or 700μg/ml (for Caco2 cells) G418 for 3 weeks.

### Semi-quantitative RT-PCR and quantitative PCR (qPCR)

Total RNA was isolated from exponentially growing cells (70%-80% confluence) by RNeasy Mini Kit (QIAGEN, Germany) according to the manufacturer’s instruction. Total RNA was converted to cDNA for semi-quantitative RT-PCR or qPCR analyses by using SuperScript® VILO™ cDNA Synthesis Kit (Invitrogen, USA). RT-PCR was done according to the manufacturer’s instructions using Platinum^®^ PCR SuperMix High Fidelity Kit (Invitrogen, USA). The qPCR analysis was performed using the Fast SYBR^®^ Green Master Mix on the ABI 7900HT® Real-Time PCR system (Applied Biosystems, USA) as previously described [27]. Changes in gene expression were represented as fold change relative to the respective control cells, and the sequences of the oligonucleotide primers used are provided in Supplementary Table 1.

### Western blot and immunofluorescence analyses

Western blot analysis was done as previously described [41] by using the following antibodies: anti-hGH (National Hormone and Peptide Program, CA); anti-E-cadherin (ab1416, Abcam); anti-Fibronectin 1 (ab2413, Abcam); anti-Occludin (ab31721, Abcam); anti-p-ERK (sc-7383, Santa Cruz); anti-ERK (sc-93, Santa Cruz), and anti-β-actin (sc-58222, Santa Cruz). Immunofluorescence analysis was performed as previously described [89], and visualized under Nikon A1R-A1 Confocal Microscope System.

### Cell proliferation and apoptosis analyses

Cell proliferation was determined using BrdU Cell Proliferation Assay Kit (Millipore) according to the manufacturer’s instructions. Cell apoptosis was determined using Caspase-Glo 3/7 Assay Kit (Promega) according to the manufacturer’s protocol.

### Cell functional assays

Functional *in vitro* assays including foci formation, colony formation in soft agar, cell growth on 2D or in 3D Matrigel, wound healing, and transwell migration and invasion assays were performed as previously described [27]. For total cell number assay, cells were seeded in a 6-well plate at a density of 5×10^3^ cells/well and cultured in 0.5% FBS or 10% FBS supplemented media as previously described [27]. Colony scattering assay was performed as described previously [90].

### Luciferase reporter assay

Luciferase reporter assays were performed using the pGL3-Basic Vector (Promega, USA) containing full length E-CADHERIN gene promoter (provided by Dr. Pandey), or pXP2 Vector containing full length FIBRONECTIN 1 gene promoter (provided by Professor Andrei Bakin from Roswell Park Cancer Institute). The promoter activities were determined by using Dual-Luciferase Reporter Assay Systems (Promega), according to the manufacturer’s instructions.

### Colonosphere formation and ALDEFLUOR assays

Colonosphere formation and ALDEFLUOR assays were carried out as previously described [51]. For colonosphere formation assay, the numbers of colonospheres were counted under a microscope after 1-2 weeks. The ALDEFLUOR assay was performed using the ALDEFLUOR™ Kit (STEMCELL Technologies, USA) according to the manufacturer’s protocol, and the fluorescence-activated cell sorting (FACs) analysis was carried out using the FACS LSR II machine (BD Biosciences, San Jose, CA).

### *In situ* hybridization (ISH) and immunohistochemistry (IHC)

The tissue samples were collected from 101 patients with colorectal cancer and 20 patients with benign colorectal disease from the First Affiliated Hospital of Anhui Medical University (Hefei, Anhui, People’s Republic of China). ISH analysis was performed with digoxin-labeled antisense oligonucleotide probes for hGH as previously described [21]. IHC analysis was performed with polyclonal anti-hGH antibody (Santa Cruz, CA) using the peroxidase-conjugated streptavidin complex method, as previously described [21]. The scoring of hGH staining was performed as described previously [21], independently by two investigators, who were blinded from the patients’ clinicopathological information.

### Xenograft analyses

Xenograft studies were performed as described previously [90]. DLD-1-vector or DLD-hGH cells (800 cells) were suspended in 125 μl Matrigel/PBS (1:1, v/v) and subcutaneously injected into the subscapular region of immunodeficient nude mice (Shanghai Slaccas Co., Shanghai). Palpable tumors were formed 20 days following injection and the tumor volumes were measured every 3-4 days. TUNEL analysis was performed as previously described [27]. For the IHC analysis, immunostaining was performed using the following antibodies: anti-Ki-67 (Angiobio & Beijing Zhongshan Jinqiao Biotechnology Co.), anti-E-cadherin (BD Biosciences) and anti-Fibronectin 1 (BD Biosciences).

### Statistics

All of the experiments in this study were performed at least three times and results from a single representative experiment is shown. All of the numerical data were expressed as mean ± standard error (SEM) of triplicate determinants. Data was analyzed by using an unpaired two-tailed *t* test with Microsoft Excel, unless otherwise stated. The correlation of hGH expression with CRC and with clinicopathological parameters of CRC patients was analyzed by Chi-squared test using SPSS.

## SUPPLEMENTARY MATERIALS FIGURES AND TABLE




