# Supplementary material for: Autocrine hGH stimulates oncogenicity, epithelial-mesenchymal transition and cancer stem cell-like behavior in human colorectal carcinoma
Source: Oncotarget. 2017 Oct 10;8(61):103900–18. doi: 10.18632/oncotarget.21812 (PMC5732775; doi:10.18632/oncotarget.21812)
Supplement: Supplementary file 1 [file oncotarget-08-103900-s001.pdf]

# Autocrine hGH stimulates oncogenicity, epithelial-mesenchymal transition and cancer stem cell-like behavior in human colorectal carcinoma

## SUPPLEMENTARY MATERIALS

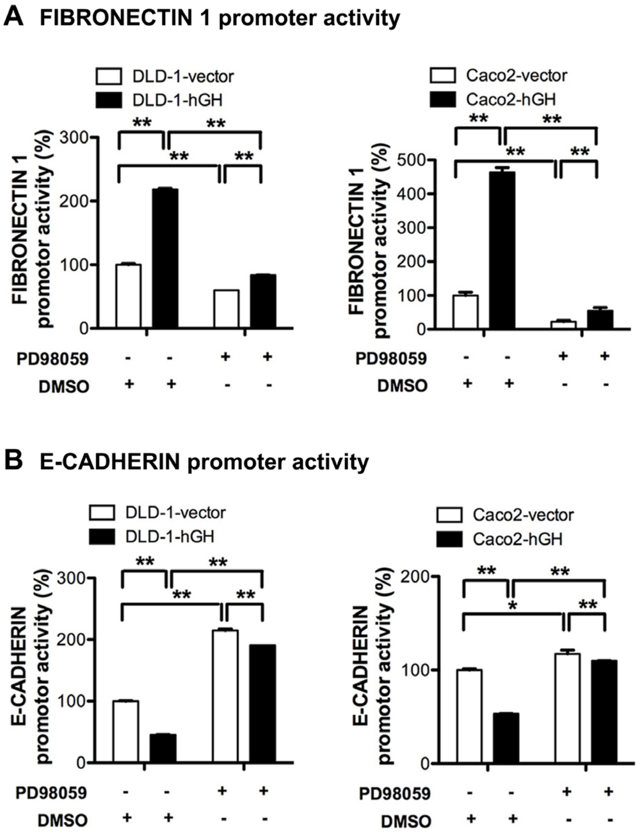


**Supplementary Figure 1: Inhibition of ERK1/2 activity significantly abrogated hGH-mediated increase in FIBRONECTIN 1 promoter activities and decrease in E-CADHERIN promoter activities in CRC cells.** Promoter activities of EMT markers **(A)** FIBRONECTIN 1 and **(B)** E-CADHERIN in DLD-1 and Caco2 stable cells ± PD98059 (20µM) were examined by promoter luciferase reporter assay. $^{*}$, $p<0.05$; $^{**}$, $p<0.01$.

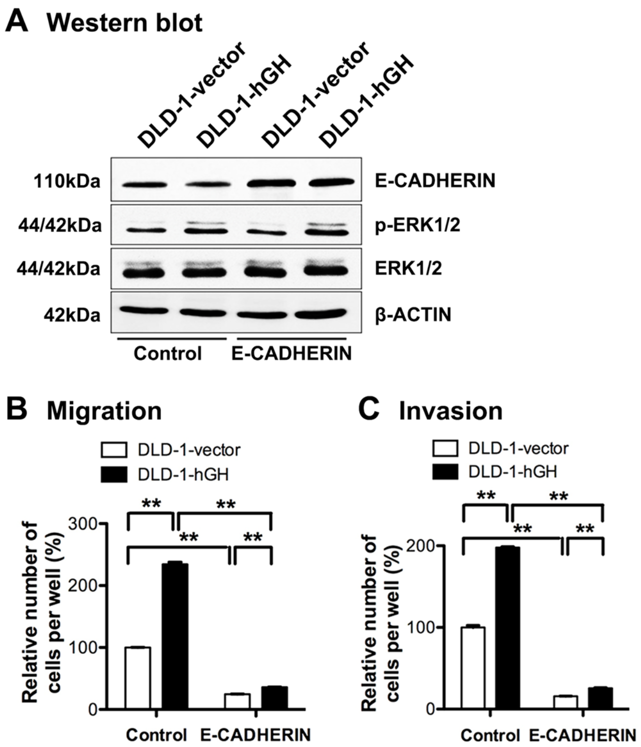


**Supplementary Figure 2: Forced expression of E-CADHERIN abrogated autocrine hGH-stimulated cell migration and invasion in DLD-1 cells.** DLD-1-vector and DLD-1-hGH cells were transfected with a vector containing E-CADHERIN gene or an empty vector as a control. **(A)** Protein expression was examined by western blot analysis. β-ACTIN was used as an input control. **(B)** Cell migration and **(C)** invasion of DLD-1 stable cells with forced expression of E-CADHERIN were evaluated using transwell assays. Cells that have migrated or invaded through the transwell membrane were stained with Hoechst 33342 and counted under a fluorescence microscope. Results were represented as percentages relative to the DLD-1-vector cells in the control group. $^{**}$, $p<0.01$.

**Supplementary Table 1: Primers used for quantitative real-time PCR**

See supplementary File 1
