# Supplementary material for: Autocrine hGH stimulates oncogenicity, epithelial-mesenchymal transition and cancer stem cell-like behavior in human colorectal carcinoma
Source: Oncotarget. 2017 Oct 10;8(61):103900–18. doi: 10.18632/oncotarget.21812 (PMC5732775; doi:10.18632/oncotarget.21812)
Supplement: Supplementary file 2 [file oncotarget-08-103900-s002.docx]

**Supplementary Table 1: Primers used for quantitative real-time PCR**

| **Gene** | **Primer sequence (5’ to 3’)** |
| --- | --- |
| *CDH1* | F: CGAGAGCTACACGTTCACGG  R: GGGTGTCGAGGGAAAAATAGG |
| *OCLN* | F: TGCCGCGTTGGTGATCTTT  R: GCCCAGGATAGCACTCACTATT |
| *CTNNA1* | F: CCATGCAGGCAACATAAACTTC  R: GGCTCCAACAGTCTCTCAACT |
| *CTNNB1* | F: CCCACTGGCCTCTGATAAAGG  R: ACGCAAAGGTGCATGATTTG |
| *CTNND2* | F: TTCATCACAGGTGCTGCGTAA  R: CCATCACACTCTCTCATCCTTCTG |
| *TWIST1* | F: GTCCGCAGTCTTACGAGGAG  R: GCTTGAGGGTCTGAATCTTGCT |
| *VIM* | F: CCTTGAACGCAAAGTGGAATC  R: GACATGCTGTTCCTGAATCTGAG |
| *FN1* | F: GGTGACACTTATGAGCGTCCTAAA  R: AACATGTAACCACCAGTCTCATGTG |
| *CDH2* | F: AGCCAACCTTAACTGAGGAGT  R: GGCAAGTTGATTGGAGGGATG |
| *SNAI1* | F: AATCGGAAGCCTAACTACAGCG  R: GTCCCAGATGAGCATTGGCA |
| *SNAI2* | F: AAGCATTTCAACGCCTCCAAA  R: GGATCTCTGGTTGTGGTATGACA |
| *FOXC2* | F: CCTCCTGGTATCTCAACCACA  R: GGTCGAGTTCTCAATCCCCA |
| *ZEB1* | F: TTACACCTTTGCATACAGAACCC  R: TTTACGATTACACCCAGACTGC |
| *ZEB2* | F: GCGATGGTCATGCAGTCAG  R: CAGGTGGCAGGTCATTTTCTT |
| *MET* | F: TGGTGCAGAGGAGCAATGG  R: CATTCTGGATGGGTGTTTCCG |
| *MMP2* | F: CAAAAACAAGAAGACATACATCTT  R: GCTTCCAAACTTCACGCTC |
| *MMP9* | F: AGACGGGTATCCCTTCGACG  R: AAACCGAGTTGGAACCACGAC |
| *MTA1* | F: GCTGTTACACCACACAGTCTT  R: GGACTCATGTTACTGCGGTTT |
| *MTA2* | F: CCGACGGCCTTATGCTCCT  R: CTGGGCCACCAGATCTTTGAC |
| *NME1* | F: CTGCAGCCGGAGTTCAAAC  R: GCAATGAAGGTACGCTCACAGT |
| *PLAU* | F: CACGCAAGGGGAGATGAA  R: ACAGCATTTTGGTGGTGACTT |
| *PLAUR* | F: AATGGCCGCCAGTGTTACAG  R: CAGGAGACATCAATGTGGTTC |
| *CD24* | F: CTCCTACCCACGCAGATTTATTC  R: AGAGTGAGACCACGAAGAGAC |
| *CD44* | F: CCCATCCCAGACGAAGACAG  R: ACCATGAAAACCAATCCCAGG |
| *KLF4* | F: CCCACATGAAGCGACTTCCC  R: CAGGTCCAGGAGATCGTTGAA |
| *SRC* | F: TGGCAAGATCACCAGACGG  R: GGCACCTTTCGTGGTCTCAC |
| *EGFR* | F: AGGCACGAGTAACAAGCTCAC  R: ATGAGGACATAACCAGCCACC |
| *NCAM1* | F: TGTGTGGTTACAGGCGAGGA  R: TTGGCGCATTCTTGAACATGA |
| *ALDH1* | F: GCACGCCAGACTTACCTGTC  R: CCTCCTCAGTTGCAGGATTAAAG |
| *WNT5A* | F: ATTCTTGGTGGTCGCTAGGTA  R: CGCCTTCTCCGATGTACTGC |
| *WNT5B* | F: GTGCAGAGACCCGAGATGTTT  R: TTGGCTCCCTCCCCTATGTAG |
| *BMI1* | F: GCTGCCAATGGCTCTAATGAA  R: TGCTGGGCATCGTAAGTATCTT |
| *LIN28A* | F: AGCGCAGATCAAAAGGAGACA  R: CCTCTCGAAAGTAGGTTGGCT |
| *NANOG* | F: TTTGTGGGCCTGAAGAAAACT  R: AGGGCTGTCCTGAATAAGCAG |
| *SOX2* | F: GCCGAGTGGAAACTTTTGTCG  R: GGCAGCGTGTACTTATCCTTCT |
| *POU5F1* | F: CTTGAATCCCGAATGGAAAGGG  R: CCTTCCCAAATAGAACCCCCA |
| *SALL4* | F: GGGCCGACACTCTGAAGAC  R: GGCTTGTTTCAAGGCATCCAG |
| *β-ACTIN* | F: GTCTGCCTTGGTAGTGGATAATG  R: TCGAGGACGCCCTATCATGG |
| *HPRT* | F: TGACACTGGCAAAACAATGCA  R: GGTCCTTTTCACCAGCAAGCT |
| *GAPDH* | F: TGCACCACCAACTGCTTAGC  R: GGCATGGACTGTGGTCATGAG |
